# Accuracy of partial coherence interferometry in patients with large inter-eye axial length difference

**DOI:** 10.1371/journal.pone.0246721

**Published:** 2021-02-09

**Authors:** Christine A. Petersen, Daniel C. Terveen, Tyler Quist, Parisa Taravati, Leona Ding, Thomas A. Oetting, Philip P. Chen

**Affiliations:** 1 Department of Ophthalmology, University of Washington, Seattle, Washington, United States of America; 2 Department of Ophthalmology, University of Iowa, Iowa City, Iowa, United States of America; Nicolaus Copernicus University, POLAND

## Abstract

**Background:**

To determine accuracy of partial coherence interferometry (PCI) in patients with large inter-eye axial eye length (AEL) difference.

**Methods:**

Patients undergoing cataract surgery at two academic medical centers with an inter-eye axial eye length (AEL) difference of > 0.30 mm were identified and were matched to control patients without inter-eye AEL difference > 0.30 mm on the basis of age, sex, and AEL. The expected post-operative refraction for the implanted IOL was calculated using SRK/T, Holladay II, and Hoffer Q formulae. The main outcome measures were the refractive prediction error and the equivalence of the refractive outcomes between the subjects and controls.

**Results:**

Review of 2212 eyes from 1617 patients found 131 eyes of 93 patients which met inclusion criteria. These were matched to 131 control eyes of 115 patients. The mean AEL was 24.92 ± 1.50 mm. The mean absolute error (MAE) ranged from 0.47 D to 0.69 D, and was not statistically different between subjects and controls. The refractive prediction error was equivalent between the cases and controls, with no significant difference between the MAE for any formula, nor in the number of cases vs. controls with a refractive prediction error of at least 0.50 D or 1.00 D.

**Conclusions:**

Among eyes in our study population, good-quality PCI data was equally accurate in patients with or without an inter-eye AEL difference > 0.30 mm. Confirmatory AEL measurements using different AEL measuring modalities in patients with a large inter-eye AEL difference may not be necessary.

## Introduction

Accurate pre-operative biometry is essential for appropriate intraocular lens (IOL) selection in cataract surgery. An axial eye length (AEL) measurement error of 0.10 mm may result in a 0.28 diopter (D) error in refractive outcome, although this value varies based on the true AEL [1].

Partial coherence interferometry (PCI) is often used as the method of choice to obtain pre-operative biometry measurements. If a difference of greater than 0.30 mm in AEL between the two eyes is measured on PCI, confirmatory AEL measurements are then performed, often with immersion A scan biometry [2]. Rajan et al. found that 24% of patients undergoing routine cataract surgery had an AEL difference of > 0.30 mm between eyes [3], suggesting that a substantial proportion of patients undergo confirmatory testing during the pre-operative evaluation. However, AEL measurements by PCI result in more accurate IOL selection than those based on ultrasound biometry [4–7].

To investigate the accuracy of IOL calculation using PCI in patients with large (> 0.30 mm) inter-eye AEL difference we compared refractive prediction error, defined as the difference between the observed and expected post-operative spherical equivalent (SE) at post-operative month one, in patients with this difference compared with a matched control group.

## Materials and methods

A retrospective review of all patients undergoing cataract surgery with IOL placement at two institutions, University of Iowa and University of Washington, for a 1-year period from 7/1/14 to 6/30/15 was performed after obtaining Institutional Review Board approval. Patients with an AEL difference of at least 0.30 mm as measured by PCI (IOLMaster 500, Carl Zeiss Meditec, Dublin, CA) were identified. To minimize the effect of external factors other than AEL measurement on postoperative refraction, we excluded eyes with signal to noise ratio <100 on PCI, any intra- or post-operative complication, AEL > 2 standard deviations from the mean of the study population, > 3 D cylinder on pre-operative refraction, toric or multifocal IOL implantation, history of refractive surgery, or cataract surgery combined with any other procedure. Eyes with best corrected visual acuity (BCVA) worse than 20/20 at post-operative month one (POM 1) were also excluded to ensure accuracy of the post-operative refraction. Control eyes with an inter-eye AEL difference of < 0.30 mm were matched for age (± 10 years), sex, and AEL (± 0.50 mm). Both eyes of a patient were included if they met the criteria, to avoid bias from exclusion of either the longer or shorter eye. We defined myopia to be an axial length of ≥ 24.80 mm.

We used the IOLMaster 500 to determine the expected post-operative refractive SE for the implanted IOL using the SRK/T and Hoffer Q formulae. Holladay IOL Consultant software (Holladay IOL Consultant, Holladay Consulting, Bellaire, TX) was used to determine the expected post-operative refractive SE for Holladay II formula. Optimized constants were used for the dataset. The IOL material was either silicone or acrylic, at the discretion of the surgeon. The observed SE based on the POM 1 refraction was obtained. A difference in observed minus expected SE of greater than 0.50 D was chosen to be clinically meaningful.

The mean absolute error (MAE) was calculated for each IOL calculation formula. The Mann-Whitney U Test was used to compare the MAE for each of the three formulae. A two-tailed Chi-Square test was used to evaluate the number of cases vs. controls whose refractive prediction error was at least 0.50 D and at least 1.00 D. We performed sub-analyses of myopes and non-myopes, and of cases with difference in AEL of 0.30–0.59 mm, 0.60–0.89 mm, and ≥ 0.90 mm using one-way ANOVA.

An Equivalence Test using ± 0.50 D as the limit of equivalence was performed to determine whether the refractive prediction error based on PCI measurements were statistically the same for cases and controls. In this statistical analysis, the null hypothesis was that the groups compared were not equivalent; a statistically significant measurement indicated that the measures were statistically equal.

The required sample size to achieve a power of 0.80 was calculated using SAS version 9.4 PROC POWER with the goal of detecting a difference of at least 0.5D in the refractive prediction error between the cases vs controls. We assumed a two-sided independent t-test with a common D of 0.50, a significant value of 0.05, and 80% power to estimate the need for 18 individuals per group (total 36) with an allocation ratio of 1:1 to provide adequate power for the analysis of equivalence.

## Results

A total of 2212 cataract surgeries of 1617 patients were reviewed; 131 eyes of 93 patients met inclusion criteria and were matched to 131 eyes of 115 control patients. The mean AEL was 24.92 ± 1.50 (range 21.49–30.17). Among pre-operative baseline characteristics, the control group had slightly better pre-operative visual acuity (Table 1). We found no significant difference between the MAE for any of the 3 formulae tested (P ≥ 0.05, Mann-Whitney U Test; Table 2). This held true in both the myopic and nonmyopic subgroups (Table 2). Furthermore, there was no difference in the MAE when subjects were stratified according to difference in AEL of 0.30–0.59 mm, 0.60–0.89 mm, and ≥ 0.90 mm between the patient’s two eyes (p = 0.62, 0.89, and 0.49 for SRK/T, Holladay II, and Hoffer Q formulae respectively, Table 3). No significant difference was found in the number of cases vs. controls with a refractive prediction error of at least 0.50 D or at least 1.00 D (P ≥ 0.05, Chi-Square test; Table 4). To determine whether enrolling both eyes of a single patient affected our results, we analyzed the MAE for cases and controls with all 3 formulae, using only first eye surgeries. The results were very similar (N = 94; p = 0.689 with SRK-T, p = 0.091 with Holladay 2, and p = 0.799 with Hoffer Q).

**Table 1:** Baseline characteristics of included cases with inter-eye axial eye length difference > 0.30 mm and controls with inter-eye axial eye length difference < 0.30 mm.

Demographic	All Cases (N = 131)	All Controls (N = 131)	p value (all cases vs all controls)	Myopic Cases (N = 65)	Myopic Controls (N = 65)	Nonmyopic Cases (N = 66)	Nonmyopic Controls (N = 66)
Age (years)	68.2 ± 8.8	67.4 ± 9.8	0.486	65.7 ± 8.6	65.0 ± 8.4	70.8 ± 8.3	69.8 ± 10.6
Pre-op BCVA (logMAR)	0.20 ± 0.17	0.16 ± 0.16	0.049	0.22 ± 0.17	0.17 ± 1.8	0.18 ± 0.18	0.14 ± 0.13
Pre-op SE, absolute value (D)	3.88 ± 3.20	3.32 ± 3.37	0.173	5.79 ± 3.32	5.15 ± 3.87	2.00 ± 1.56	1.53 ± 1.19
*Pre-op SE (D)*	*-3*.*17 ± 3*.*91*	*-2*.*58 ± 3*.*98*	*0*.*220*	*-5*.*56 ± 3*.*69*	*-4*.*98 ± 4*.*09*	*-0*.*82 ± 2*.*41*	*-2*.*14 ± 1*.*93*
Pre-op AEL (mm)	24.93 ± 1.54	24.79 ± 1.46	0.916	26.15 ±1.10	26.06 ± 1.09	23.74 ± 0.79	23.79 ± 0.72
SNR for PCI Measurements	274.7 ± 130.9	259.1 ± 117.7	0.347	290.1 ± 131.0	251.1 ± 120.1	257.5 ± 129.7	267.2 ± 115.7
Inter-Eye AEL Difference (mm)	0.75 ± 0.58	0.17 ± 0.31	0.000*	0.77 ± 0.65	0.16 ± 0.08	0.74 ± 0.50	0.18 ± 0.43
ACD (mm)^†^	3.36 ± 0.42	3.23 ± 0.49	0.091				
% Acrylic IOL	90.0%	88.5%					
% Right Eye	49.6%	47.3%					
% Shorter Eye	48.9%	52.7%					

Values presented as mean ± standard deviation. SE = spherical equivalent, D = diopters; AEL = axial eye length; SNR = signal to noise ratio; PCI = partial coherence interferometry; ACD = anterior chamber depth; Myopia = AEL ≥ 24.8 mm.

^†^78 case and 79 control eyes had ACD data.

Results are given as mean ± standard deviation.

*Denotes a statistically significant p value.

**Table 2:** Mean numerical error, median absolute error, and mean absolute error (MAE) for the SRK/T, Holladay II, and Hoffer Q formulae.

IOL Calculation Formula	Mean Numerical Error (D)		Median Absolute Error (D)		Mean Absolute Error (D)		p value for Mean Absolute Error
	Cases	Controls	Cases	Controls	Cases	Controls	
SRK/T							
All (N = 131)	0.29 ± 0.56	0.38 ± 0.54	0.38	0.42	0.47 ± 0.41	0.51 ± 0.42	0.453
Myopes (N = 65)	0.32 ± 0.53	0.42 ± 0.56	0.37	0.42	0.47 ± 0.40	0.52 ± 0.47	0.535
Nonmyopes (N = 66)	0.26 ± 0.58	0.35 ± 0.52	0.44	0.45	0.48 ± 0.42	0.50 ± 0.37	0.589
Holladay II							
All (N = 131)	0.28 ± 0.59	0.39 ± 0.38	0.39	0.53	0.51 ± 0.45	0.60 ± 0.47	0.074
Myopes (N = 65)	0.37 ± 0.54	0.45 ± 0.61	0.35	0.60	0.49 ± 0.43	0.62 ± 0.46	0.063
Nonmyopes (N = 66)	0.19 ± 0.63	0.33 ± 0.55	0.46	0.48	0.52 ± 0.44	0.57 ± 0.46	0.610
Hoffer Q							
All (N = 131)	0.47 ± 0.74	0.53 ± 0.55	0.51	0.58	0.61 ± 0.48	0.63 ± 0.45	0.682
Myopes (N = 65)	0.59 ± 0.56	0.63 ± 0.56	0.53	0.68	0.65 ± 0.49	0.69 ± 0.49	0.497
Nonmyopes (N = 66)	0.35 ± 0.62	0.43 ± 0.53	0.50	0.55	0.55 ± 0.44	0.54 ± 0.39	0.976

D = diopters; myopia = AEL ≥ 24.80 mm.

Results of the Mann-Whitney U Test for the MAE of cases with inter-eye axial eye length difference > 0.30 mm versus controls with inter-eye axial eye length difference < 0.30 mm. Results are given as mean ± SD.

**Table 3:** Sub-analysis of cases stratified by difference in axial eye length between the two eyes of each case patient of 0.30–0.59 mm, 0.60–0.89 mm, and ≥ 0.90 mm.

IOL Calculation Formula	Mean Absolute Error (D)		p value
	Cases	Controls	
SRK/T			
ΔAEL 0.30 mm—0.59 mm (N = 71)	0.49 ± 0.48	0.512± 0.35	0.208
ΔAEL 0.60mm—0.89 mm (N = 28)	0.44 ± 0.31	0.63 ± 0.58	0.285
ΔAEL ≥ 0.90 mm (N = 32)	0.48 ± 0.32	0.40 ± 0.38	0.144
Holladay II			
ΔAEL 0.30mm—0.59 mm (N = 71)	0.54 ± 0.48	0.61 ± 0.35	0.054
ΔAEL 0.60 mm—0.89 mm (N = 28)	0.47 ± 0.36	0.76 ± 0.70	0.226
ΔAEL ≥ 0.90 mm (N = 32)	0.46 ± 0.40	0.41 ± 0.35	0.596
Hoffer Q			
ΔAEL 0.30 mm—0.59 mm (N = 71)	0.65 ± 0.53	0.66 ± 0.38	0.276
ΔAEL 0.60 mm—0.89 mm (N = 28)	0.54 ± 0.34	0.67 ± 0.60	0.660
ΔAEL ≥ 0.90 mm (N = 32)	0.55 ± 0.41	0.46 ± 0.40	0.294

D = diopters; ΔAEL = difference in axial eye length between two eyes of case patient; N = number in each group.

* Mann-Whitney U Test

Results are given as mean ± SD.

**Table 4:** Results of the Chi-Square Test for the post-operative month one spherical equivalent of cases with inter-eye axial eye length difference of > 0.30 mm versus controls with inter-eye axial eye length difference < 0.30 mm.

IOL Calculation Formula	N (%) with MAE within 0.5 D of predicted refraction		p value	N (%) with MAE within 1.0 D of predicted refraction		p value
	Cases	Controls		Cases	Controls	
SRK/T						
All (N = 131)	88 (67)	81 (62)	0.705	122 (93)	116 (89)	0.310
Myopes (N = 65)	45 (69)	42 (65)	0.716	60 (92)	58 (89)	0.545
Nonmyopes (N = 66)	43 (65)	39 (59)	0.861	62 (94)	58 (88)	0.411
Holladay II						
All (N = 131)	86 (66)	68 (52)	0.227	117 (89)	112 (85)	0.078
Myopes (N = 65)	45 (69)	30 (46)	0.014*	56 (86)	56 (86)	0.250
Nonmyopes (N = 66)	41 (62)	38 (58)	0.727	61 (92)	56 (85)	0.170
Hoffer Q						
All (N = 131)	73 (56)	61 (47)	0.702	115 (88)	109 (83)	0.241
Myopes (N = 65)	36 (55)	29 (45)	0.720	54 (83)	51 (78)	0.292
Nonmyopes (N = 66)	37 (66)	33 (50)	0.862	61 (92)	58 (88)	0.572

SE = spherical equivalent

Results are given as mean ± SD. Myopia = AEL ≥ 24.80 mm.

*Denotes a statistically significant p value.

Each IOL formula was separately analyzed for refractive differences. In the myopic group using the Holladay II formula, there were significantly more patients without a large inter-eye AEL difference who had a refractive outcome greater than 0.50 D different than expected. None of the other formulae showed this difference for either myopes or nonmyopes (Table 4). Equivalence testing found that the refractive prediction error was equivalent between cases with an inter-eye AEL difference of > 0.30 mm and controls without such a difference for each of the 3 formulae used (p = 0.000, Table 5).

**Table 5:** Equivalence Testing of the MAE in cases with inter-eye axial eye length difference of > 0.30 mm and controls with inter-eye axial eye length difference of < 0.30 mm using 3 different intraocular lens formulae.

IOL Calculation Formula	Mean Absolute Error (D ± SD)		Difference in Mean Absolute Error for Cases vs Controls (D ± SE)	95% Confidence Interval	p value
	Cases	Controls			
SRK/T	0.47 ± 0.41	0.51 ± 0.42	0.04 ± 0.05	-0.047, 0.12	0.000*
Holladay II	0.51 ± 0.44	0.60 ± 0.46	0.09 ± 0.06	-0.003, 0.19	0.000*
Hoffer Q	0.61 ± 0.48	0.63 ± 0.45	0.02 ± 0.06	-0.078, 0.12	0.000*

D = diopters; SD = standard deviation

A significant P value indicates the results from each group are statistically equal.

*Denotes a statistically significant p value.

Examination of refractive accuracy revealed that 47–67% of study eyes were within 0.50 D and 83–93% were within 1.00 D of the refractive target, depending on the IOL calculation formula used.

## Discussion

Determining the accuracy of PCI in patients with a large inter-eye AEL difference is vitally important in achieving optimum refractive outcomes. The Royal College of Ophthalmologists Cataract Surgery Guidelines suggest that confirmatory testing should be considered if the AEL difference between the two eyes is at least 0.30 mm [2]. We found good-quality PCI to be equally accurate in eyes with or without a large inter-eye AEL difference. Our findings suggest that while confirmatory testing is probably wise in such patients, repeat PCI may be an acceptable mode of confirmatory testing rather than use of alternate biometry methods such as immersion A-scan. This has practical implications in the clinical setting, since a confirmatory measurement with a separate instrument requires additional time, may be uncomfortable for the patient, and increases the expense to the practice and the health care system of performing cataract surgery.

We found that 267 of 1617 (16.5%) patients had an inter-eye AEL difference of at least 0.30 mm. Our findings are similar to those reported in other studies. In a study of 14,016 eyes of 7,008 patients, Knox Cartwright et al. found that 17.5% of patients had AEL asymmetry of at least 0.30 mm [8]. Rajan et al. studied 1379 patients undergoing uneventful bilateral cataract surgery, in which 24% had an inter-eye AEL difference of > 0.30 mm. They also noted that AEL differences increased with increasing AEL, and that differences in study populations may contribute to discrepancies in rates of AEL between-eye differences [3]. Various studies have found mean inter-eye AEL differences in the range of 0.21 ± 0.35 mm to 0.34 ± 0.70 mm [8–10].

We excluded eyes with AEL ± 2 SD from the mean, which may explain why the proportion of patients with a large AEL difference was slightly lower in our study compared to some others in the literature. Nonetheless, a large minority of patients can be expected to have an inter-eye AEL difference of at least 0.30 mm, and the ability to obtain accurate pre-operative measurements in these patients is crucial [3, 8].

Kansal et al. [11] reported on the effect of inter-eye AEL difference on refractive outcome after cataract surgery in 1458 eyes of 729 patients; the risk of having a refractive outcome >0.5 D from goal increased with increasing inter-eye AEL difference, with an odds ratio of 1.6 for inter-eye AEL difference of >0.30 mm and of 1.4 for a difference of 0.20 mm. Our study design was different from Kansal et al. (case control vs cohort) because we specifically sought to determine the effect of AEL difference >0.30 vs <0.30 mm; our control population had a mean AEL difference of almost 0.20 mm. In addition, the study populations differed in several ways, which may explain the difference in findings between the studies. The mean axial length was longer in our study, and we had more exclusion criteria, to reduce the likelihood that the postoperative refraction could be affected by non-AEL factors.

Our sub-analysis of myopic vs non-myopic eye provides important insight. Myopic eyes have previously been shown to have a higher rate of refractive surprise, with lower proportions of eyes falling within 0.50 D or 1.0 D of the predicted refractive outcome compared to non-myopic eyes [3, 11]. Also, the asymmetry in axial length between two eyes of a patient tends to increase with increasing axial length [3]. This means that myopes are more likely to have a difference in AEL >0.30 mm between the two eyes compared to non-myopes. The fact that myopic eyes with inter-eye AEL > 0.30 mm compared to their fellow eye in our study did not have significantly higher post-operative MAE compared to their AEL-matched controls suggests that although myopic eyes may indeed be at higher risk for refractive surprise, their PCI biometry readings are equally accurate whether or not they have a large inter-eye AEL difference.

Limitations of this study include its retrospective nature and the use of non-optimized third generation IOL power formulae. Our percentage of patients with a refractive outcome within 0.50 D of the predicted target ranged from 47–67%, and 83–93% were within 1.00 D; given our longer mean AEL, these results are not surprising, with the SRK T formula delivering the highest accuracy [12]. Our findings are similar to those reported recently elsewhere [13].

In summary, our study demonstrates that the accuracy of good-quality PCI biometry measurements is not significantly different in patients with a large inter-eye AEL difference compared to matched controls with a smaller inter-eye AEL difference. This finding held true for both myopes and non-myopes in sub-analysis. Confirmation of large inter-eye AEL differences should be performed to reduce the risk of refractive surprises after phacoemulsification, but such a confirmation may be performed with the same PCI instrument and the resulting measurements can be considered reliable. Further study in this important area is warranted.

## Supporting information

S1 Data De-identified data. (XLSX)
